# The association between pupillary responses and axial length in children differs as a function of season

**DOI:** 10.1038/s41598-024-51199-0

**Published:** 2024-01-05

**Authors:** Marielle G. Reidy, Andrew T. E. Hartwick, Donald O. Mutti

**Affiliations:** The Ohio State University College of Optometry, Columbus, USA

**Keywords:** Paediatrics, Medical research

## Abstract

The association between pupillary responses to repeated stimuli and adult refractive error has been previously demonstrated. This study evaluated whether this association exists in children and if it varies by season. Fifty children aged 8–17 years (average: 11.55 ± 2.75 years, 31 females) with refractive error between + 1.51 and − 5.69 diopters (non-cycloplegic) participated (n = 27 in summer, and n = 23 in winter). The RAPDx pupilometer measured pupil sizes while stimuli oscillated between colored light and dark at 0.1 Hz in three sequences: (1) alternating red and blue, (2) red-only, and (3) blue-only. The primary outcome was the difference in pupillary responses between the blue-only and red-only sequences. Pupillary constriction was greater in response to blue light than to red for those with shorter eyes in summer (β = − 9.42, P = 0.034) but not in winter (β = 3.42, P = 0.54). Greater constriction comprised faster pupillary escape following red light onset and slower redilation following stimulus offset of both colors (P = 0.017, 0.036, 0.035 respectively). The association between axial length and children’s pupillary responses in summer, but not winter may be explained by greater light-associated release of retinal dopamine in summer. Shorter eyes’ more robust responses are consistent with greater light exposure inhibiting axial elongation and reducing myopia risk.

## Introduction

While extensive research has been done on myopia, the mechanism behind its development and progression is still not entirely clear. Understanding myopia is of concern, as its prevalence is rapidly rising, growing from around 25% in 1971-2 to approximately 33% in 1999–2004 in the United States^1,2^. Globally, myopic prevalence is expected to reach nearly 50% by the year 2050, with many regions reaching or exceeding 60%^3^. Though the optical blur caused by myopia can be managed with corrective lenses, such as glasses or contact lenses, or with refractive surgery, the comorbidities associated with myopia, including cataracts, glaucoma, and retinal pathology resulting from excessive ocular enlargement, make this rising prevalence especially concerning^4–6^. While there is no proven treatment that will prevent the development of myopia, its onset may be delayed and incidence reduced through spending more time outdoors or treatment with low-dose atropine^7–9^.

Certain behaviors have been linked to myopia development, and these include increased duration of near work, time spent outdoors, and engagement in sports activities. Multiple cross-sectional studies have confirmed a correlative relationship between time spent doing near activities and the presence of myopic refractive error^10,11^. Despite this strong correlation, a causal role for increased near work in myopia onset has not been firmly established^8,12–14^. In addition to spending more time on near tasks, young myopes also spend less time playing sports outdoors^15^, and children who spend more time outdoors are 10% less likely to be myopic^16^. Longitudinal studies have established that, unlike near work, spending time outdoors is protective against the onset of myopia^7,8,12^, and randomized controlled trials further support this relationship^17,18^. Clinical studies have shown mixed results when looking at this relationship once a child is myopic, making it unclear if time outdoors is protective against progression in young myopes^13,17,19^.

Exposure to bright light and its associated rise in retinal levels of dopamine^20^, a neuromodulator that has been shown in animal studies to slow eye growth^21,22^ (see Brown et al.^23^ for a recent review)^23^, has been hypothesized as a mechanism for how time outdoors might delay or prevent myopia onset^15^. Simulated outdoor light levels demonstrated a protective effect against experimental myopia in the laboratory in both chicks and rhesus monkeys, and this effect was mitigated by intravitreal injection of dopamine antagonists^24–27^. Potentially analogous results were found in a study looking at the effect of accummulated daylight hours on refractive error in humans^28^. Additionally, both myopic progression and axial elongation are slower in the summer months, when exposure to bright light is higher^28–30^.

Intrinsically photosensitive retinal ganglion cells (ipRGCs) may contribute to the rise in retinal dopamine levels that is evoked by exposure to bright light. Intraretinally, ipRGCs drive dopamine release through their synaptic connections with sustained-firing dopaminergic amacrine cells^31^. IpRGCs contain the photopigment melanopsin, which has a peak sensitivity to light wavelengths around 480 nm, and these photoreceptors are capable of directly responding to light^32^. These cells project to many brain centers, including the suprachiasmatic nucleus, where they regulate circadian rhythm synchronization. They also project to the olivary pretectal nucleus where they contribute to the pupillary response to light^33,34^. The sustained and persistent pupillary constriction that occurs after exposure to bright, short-wavelength blue light stimuli, relative to that evoked by long-wavelength stimuli of similar irradiances, is typically used to assess melanopsin-driven pupillary responses. In prior work, greater short-term light exposure was associated with an increase in melanopsin-driven pupillary responses in children, indicating that the contribution of these photoreceptors to pupil constriction may be modifiable by environmental light levels^35^. Perhaps contradictorily, these same pupillary responses are reported to be potentiated in the winter months, when light exposure is lower^36^, suggesting seasonal factors may affect the relationship between long- and short-term light exposure and melanopsin’s influence on pupillary responses.

The change in melanopsin-driven pupillary responses elicited using a specific protocol that utilizes repeated light flashes has been shown to be related to refractive error in adults^37^. The current study sought to understand whether a relationship between this adaptive change in melanopsin-driven pupil responses and refractive error exists in children and whether any effect is modified by season of the year.

## Methods

Research was conducted in accordance with the tenets of the Declaration of Helsinki and the study protocol was approved by the Institutional Review Board at the Ohio State University. Participants aged 8–17 years were recruited at the Center for Science and Industry (COSI), a children’s science museum in Columbus, OH. After being informed of the study purposes and risks, all participants signed written assent forms and at least one parent signed written consent forms. Participants had monocular visual acuity of 20/25 or better in each eye as measured with a Bailey-Lovie chart at 20 feet. Exclusion criteria were any ocular disease, including strabismus and amblyopia, history of myopia control therapy, or history of systemic diseases such as diabetes, Marfan, or Down syndrome due to their association with higher myopic refractive errors^38^. Once eligibility was confirmed, participants underwent a testing protocol consisting of pupillometry measurements with the RAPDx pupillometer (Konan Medical; Irvine, CA), non-cycloplegic refractive error measurements with the Grand Seiko WR-5100K autorefractor (AIT Industries; Bensenville, IL) utilizing a Badal track and a + 6.50 D lens to relax accommodation, and axial length measurements with the IOLMaster (Carl Zeiss Meditec; Dublin, CA). Up to ten autorefractor and up to five axial length measurements were averaged for each eye. The average of the two eyes was then used for analysis.

**Figure 1:**
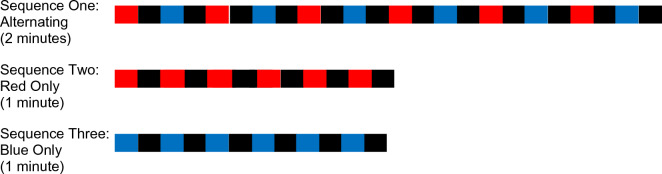
Pupillometry testing sequence performed with the RAPDx pupillometer.

With the room lights dimmed, but without any prior dark adaptation, the participants viewed red and blue light stimuli that oscillated with darkness at 0.1 Hz in a square wave pattern (Fig. 1). Participants were shown 5 seconds of red light, followed by five seconds of darkness, then five seconds of blue light, and another five seconds of darkness. This sequence repeated for 2 minutes, resulting in six stimuli of each color. Participants were then shown the second sequence: 5 seconds of red light, followed by 5 seconds of darkness, alternating between red light and darkness for one minute, resulting in six red stimuli. The final sequence showed participants five seconds of blue light, followed by 5 seconds of darkness, alternating for a total of one minute for six blue stimuli. The three sequences together with short breaks between comprised 5 minutes of testing. Pupillometry data were exported and binned into quarter-second intervals for ease of data handling.

Prior to data analysis, the pupillometry data were screened to remove non-physiological responses, which can occur due to errors in pupil capture during blinks and brief eye movements. For periods of pupil constriction, data points were removed if they showed a decrease in size more than 1.41 mm in one quarter-second interval; for periods of pupil dilation, data points were removed if they showed an increase in size more than 0.465 mm in one quarter-second interval^39^. To reduce variability in the data due to inter-individual differences in baseline pupil size, the pupillometry data were normalized. For each subject, the smallest pupil throughout the entire protocol was set as the minimum and the largest as the maximum. Using the following formula, where “pupil diameter” indicates the pupil size at the data point being normalized, the largest, most dilated pupil was normalized to equal to 0 and the smallest, most constricted pupil was normalized to equal to 1.

$$Normalized \,\,Pupillary \,\,Constriction=\frac{Maximum \,\,Diameter-Pupil \,\,Diameter}{Maximum \,\,Diameter-Minimum \,\,Diameter}.$$

**Figure 2:**
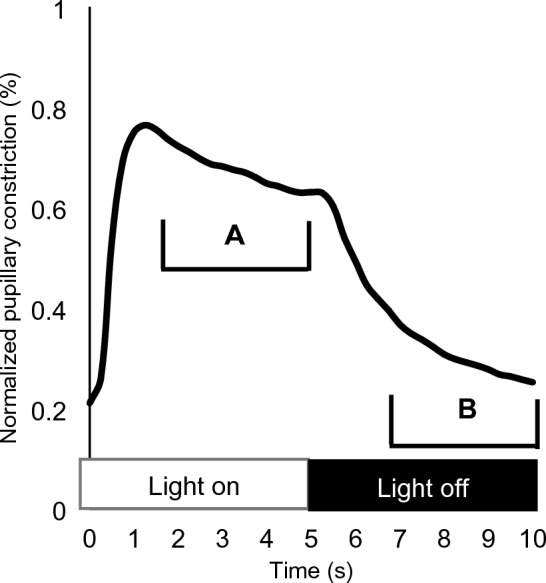
Sample of the normalized pupil constriction (y-axis) during a single pulse of light stimulation. A y-value of 0 represents a subject’s maximum dilation; a y-value of 1 represents maximum constriction throughout the entire protocol. The entire pulse lasts 10 s (x-axis), with the light on for seconds 1–5 and off for seconds 6–10. Brackets mark the portions of the normalized pupil constriction fit to the exponential decay function for calculation of A) constriction decay (i.e. pupillary escape; seconds 2–5) and B) dilation decay (seconds 7–10). The decay coefficient for corresponding portions for all six pulses of light during the single color sequence were averaged to calculate the final variable of each color of light for both constriction and dilation.

Normalized pupillary constrictions for the right and left eyes were then averaged for every 0.25 s interval. The normalized pupil data were used to calculate pupillary outcome variables as in the previous analysis of adults^37^. Table 1 provides a comprehensive list and descriptions of all pupillary outcome variables. The constriction and dilation decay variables are further described in Fig. 2.

**Table 1:** Descriptions of each pupil outcome variable and its calculation.

Variable name	Description
Alternating red (AltRed) or Alternating blue (AltBlue)	Mean normalized pupillary constriction during red or blue stimulus onset and offset in the initial alternating sequence
Red-Only or Blue-Only	Mean normalized pupillary constriction during red or blue stimulus onset and offset in the respective single color sequence
Blue minus Red	Mean of the difference in normalized pupillary constriction at corresponding time points for single color sequences
∆Red or ∆Blue	Mean of the difference in normalized pupillary constriction at corresponding time points during respective color stimulation during the single color and alternating sequence (Red-Only minus AltRed or Blue-Only minus AltBlue)
Red constriction decay or Blue constriction decay	Mean of the exponential decay coefficient (b) for the function ${e}^{bt}$ fit to seconds 2 through 5 of each pulse of respective color stimulus onset during single color sequences
Red dilation decay or Blue dilation decay	Mean of the exponential decay coefficient (b) for the function ${e}^{bt}$ fit to the last three seconds of dilation following respective color stimulus offset of each pulse during the single color sequences

Data were analyzed using SPSS software (IBM Corporation, v. 24). Participants tested between June and August were assigned to summer and those tested in January through March were assigned to winter. No participants were tested in April through May or September through December. Univariate analysis of variance analyzed the relationship between axial length and Blue minus Red in the summer and winter months separately, accounting for sex and age. Multivariate analysis of variance with a backwards stepwise modeling approach analyzed axial length as a function of all pupillary outcomes, accounting for sex and age, in separate models for the summer and winter months.

## Results

Table 2 provides the mean and standard deviation for age, spherical equivalent refractive error (SEQ), axial length (AL), maximum and minimum pupil size in millimeters, and pupil amplitude (difference between maximum and minimum pupil size) in millimeters. Male participants had longer axial lengths and greater pupil amplitudes than female participants (P = 0.005 and 0.040 respectively, independent samples t-tests, equal variances assumed). SEQ, maximum pupil size, and minimum pupil size did not differ between male and female participants (p-values between 0.058 and 0.94). None of these variables differed between summer and winter months (p-values between 0.17 and 0.97).

**Table 2:** Descriptive statistics for age, refractive error, axial length, and pupil size before normalization for all participants, and broken down by season and sex.

	All participants	Summer	Winter	Female	Male
N	50	27	23	31	19
Age (years)	11.55 (2.75)	11.64 (3.03)	11.44 (2.46)	11.57 (2.70)	11.51 (2.92)
SEQ (D)	− 0.62 (1.44)	− 0.58 (1.65)	− 0.66 (1.17)	− 0.56 (1.24)	− 0.72 (1.74)
AL (mm)	23.51 (1.07)	23.50 (0.97)	23.51 (1.20)	23.18 (0.94)*	24.04 (1.09)*
Min pupil size (mm)	2.55 (0.40)	2.55 (0.30)	2.55 (0.50)	2.53 (0.43)	2.58 (0.35)
Max pupil size (mm)	6.58 (0.67)	6.48 (0.54)	6.69 (0.80)	6.44 (0.72)	6.81 (0.53)
Pupil amplitude (mm)	4.03 (0.55)	3.93 (0.43)	4.14 (0.65)	3.90 (0.60)*	4.23 (0.39)*

Values are given as mean (SD). Significant difference in means denoted with *.

Table 3 provides the mean and standard deviation for the normalized pupillary outcome measures as well as comparisons between red and blue variables. On average, AltBlue was greater than AltRed and Blue-Only was greater than Red-Only, indicating that pupillary constriction was greater in response to blue than to red in both sequences (P < 0.001 for both), with no significant effects of season or sex on any alternating or single color pupillary responses. When compared to pupillary responses during the alternating sequences, pupils constricted more during blue-only stimulation in contrast to a slight decrease in pupillary constriction during red-only stimulation. As a result, ΔBlue was significantly greater than 0 (P < 0.001), ΔRed was significantly less than 0 (P = 0.018), and ΔBlue was greater than ΔRed (P < 0.001). Only ∆Blue differed by sex; female participants had larger ∆Blue values than male participants (P = 0.031, independent samples t-test, equal variances assumed). Red constriction decay was more negative than Blue constriction decay, indicating that pupillary escape was faster during red stimulation compared to blue (P < 0.001). Red dilation decay was also more negative than Blue dilation decay, indicating that the rate of redilation was faster following red light offset compared to blue (P < 0.001).

**Table 3:** Differences in pupillary outcomes between red and blue stimuli were statistically significant.

Pupillary outcome	Blue stimulus	Red stimulus	P-value
AltBlue vs. AltRed	0.60 (0.048)	0.57 (0.059)	< 0.001
Blue-Only vs. Red-Only	0.66 (0.055)	0.56 (0.071)	< 0.001
ΔBlue vs. ΔRed	0.052 (0.040)	− 0.015 (0.045)	< 0.001
Constriction decay Blue-Only vs. Red-Only	− 0.020 (0.024)	− 0.043 (0.029)	< 0.001
Dilation decay Blue-Only vs. Red-Only	− 0.12 (0.047)	− 0.16 (0.066)	< 0.001

Values are given as mean value (SD).

The effects of stimulus color on pupillary responses varied by season (Table 4). ΔRed was not significantly different from 0 in winter (P = 0.52) but was significantly less than 0 in summer (P = 0.017), while ΔBlue was significantly greater than 0 in both seasons (P < 0.001). The difference in adaptation between earlier alternating pulses and later single color pulses was therefore greater for blue than for red; participants tested in the summer had larger differences between ΔBlue and ΔRed than those tested in winter (P < 0.001). While pupillary responses to single color stimuli were unaffected by season, participants tested in the summer had greater Blue minus Red values than those tested in winter, representing larger differences between Blue-Only and Red-Only (P = 0.007).

**Table 4:** Differences in pupillary outcomes between seasons were statistically significant.

Pupillary outcome	Summer	Winter	P-value
Blue minus Red	0.11 (0.048)	0.073 (0.046)	0.007
ΔBlue minus ΔRed	0.084 (0.039)	0.048 (0.034)	0.001

Values are given as mean value (SD).

**Figure 3:**
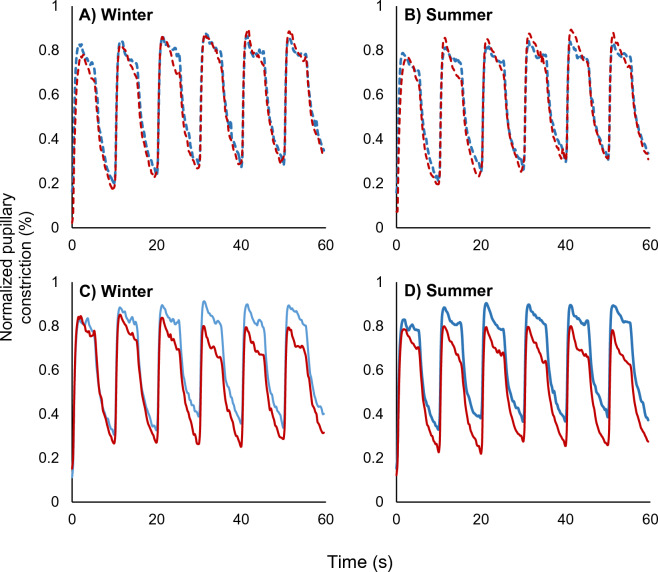
Normalized pupillary constriction (y-axis), averaged for all six pulses of each color (sixty seconds each, x-axis), during the alternating sequence (dashed lines) in the winter (**A**) and summer (**B**) and the single color sequences (solid lines) in the winter (**C**) and summer (**D**).

The greater pupillary constriction in response to the blue-only sequence compared to the red-only sequence, and the accentuation of that greater pupillary constriction in summer compared to winter, is depicted in Fig. 3. The results for the two colors are superimposed so that the first pulse of red overlaps with the first pulse of blue for easier comparison. During the alternating protocol, pupillary responses to red and blue stimulation were not affected by season (P = 0.51; Fig. 3, panels A and B). However, Fig. 3C in winter shows the greater pupillary constriction to the blue-only sequence compared to a decreasing pupillary constriction to the red-only sequence developing across the six pulses. Figure 3D shows this effect to a greater extent, with a larger gap between the responses to the red-only and blue-only stimuli in the summer (P = 0.007; Table 4).

**Figure 4:**
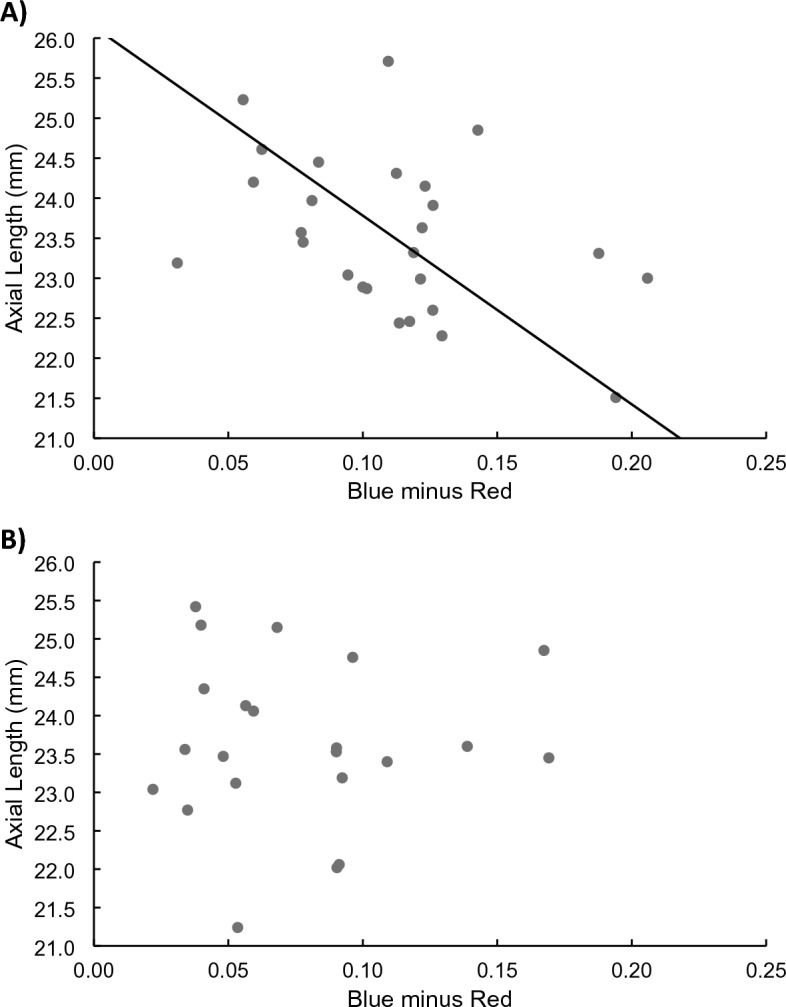
Axial length as a function of the age- and sex-adjusted Blue minus Red pupillary response, in the (**A**) summer months and (**B**) winter months.

Spherical equivalent refractive error was not normally distributed (Shapiro–Wilk test statistic = 8.65, P < 0.001) while axial length was (test statistic = 9.87, P = 0.84). Therefore, analyses primarily focused on axial length. Associations with axial length could be expected to be relevant given that axial length was significantly correlated with refractive error in each season (R^2^ = 0.43 in winter, 0.44 in summer, P < 0.001 for both). We explored whether axial length was associated with Blue minus Red in separate analyses for summer and for winter; Blue minus Red was preferred compared to ∆Blue minus ∆Red due to the lack of difference in pupillary constriction between seasons during the alternating protocol. In a multivariate analysis of variance adjusting for age and sex, axial length was significantly associated with Blue minus Red in the summer months (P = 0.034), but not in the winter months (P = 0.54). In the summer, the regression between age- and sex-adjusted Blue minus Red and axial length was significant (β = − 9.42, P = 0.034, R^2^ = 0.16; Fig. 4A), while there was no significant association between the two in the winter months (β = 3.42, P = 0.54, R^2^ = 0.010; Fig. 4B). Models were also run with right eye, longer eye, and shorter eye axial length instead of the averaged right and left eye axial length in the reported model with similar results (data not shown).

The larger gap in Fig. 3D between pupillary responses to blue and red in summer appears mostly due to greater pupillary escape in response to red and slower redilation in response to blue. These features were evaluated in further detail along with other candidate variables in multiple regression models using backwards selection (decay coefficients for red and blue, both constriction and dilation, ΔBlue, and ΔRed). Blue minus Red was not retained in a final model in either season by this process. Several features of the pupillary response were significantly associated with axial length in the summer months in the final model shown in Table 5: Red constriction decay, Blue dilation decay, Red dilation decay, and ∆Red, (P = 0.017, 0.035, 0.036, and 0.008 respectively). In the summer, ∆Red showed a positive relationship with axial length, indicating that shorter eyes showed a greater reduction in pupillary constriction during the red-only stimuli compared to the alternating red stimuli. Axial length also showed a positive relationship with Red constriction decay in the summer, indicating that shorter eyes had faster pupillary escape when looking at the red light stimulus. Axial length had a negative relationship with both Red dilation decay and Blue dilation decay in the summer. Following light stimulus offset of either color, shorter eyes re-dilated more slowly than longer eyes. In the winter months only Red dilation decay was retained in the final model shown in Table 5 (P = 0.008), indicating that shorter eyes re-dilated faster than longer eyes following red light stimulus offset in both seasons. To protect against over-modelling, the same variables were assessed via a forward selection approach. The models converged to the same final model in both summer and winter regardless of which modelling approach was taken.

**Table 5:** Final multivariate ANOVA models of age- and sex-adjusted axial length in the summer and winter.

	Summer		Winter	
	β	P-value	β	P-value
Red constriction decay	12.05	0.017	–	–
Blue dilation decay	− 9.48	0.035	–	–
Red dilation decay	− 7.20	0.036	− 7.15	0.008
∆Red	9.28	0.008	–	–

Backwards modeling of all pupillary outcome measures revealed red dilation decay, blue dilation decay, red constriction decay, and ∆red as the significant variables for the final summer model. Only red constriction decay remained significant in the final model for winter. R^2^ for the models were 0.49 and 0.46 in summer and winter, respectively. When only age and sex were used to predict axial length in summer and winter, R^2^ = 0.08 and 0.25, respectively.

This final model is different from the model in adult participants, partially due to inclusion of new pupillary measures being evaluated^37^. To explore whether this more detailed analysis in children applied to the previous adult study, univariate analysis of variance was used to analyze Red constriction decay for the final pulse of the red-only sequence and Blue dilation decay in the blue-only sequence for the adult participants. Both were significantly associated with SEQ, which was the major variable of interest in the adult study (β =  − 19.2, P = 0.009; and β = 15.6, P = 0.006; respectively). The signs are reversed appropriately compared to Table 5 given that the current study analyzed axial length and the previous study of adults used refractive error. This correspondence with the adult study further validates the detailed model against the possibility of over-modelling.

Removing all pupillary measures from the model with axial length provides a perspective on their ability to account for variance in axial length compared to the demographic variables of age and sex alone. The pupillary measures accounted for the majority of the variance in axial length during the summer months as the adjusted R^2^ was only equal to 0.08 for age and sex compared to 0.49 with the pupillary outcomes included. Age and sex were more strongly associated with axial length in the winter months, accounting for a large portion of the variance in the winter model with an R^2^ = 0.25. Adding the decay coefficient for dilation in response to red to the model including axial length, age and gender increased the variance explained to 0.46, a statistically significant but smaller increase in variance explained by the pupillary outcomes in the winter than during the summer.

## Discussion

Pupillary responses to oscillating red- and blue-light stimuli differed as a function of color, season, and axial length in children aged 8 to 17 years. Pupillary constriction was greater in response to blue than to red during alternating color pulses, with even greater constriction following additional repeated blue-only pulses. Pupillary escape during the light pulses was faster for the red compared to blue and pupil redilation was slower following blue light offset compared to red. These responses are consistent with greater contribution of melanopsin-driven input that results in more sustained pupil constriction during and following exposure to blue stimuli relative to comparably bright red stimuli^35,40–43^. The greater pupillary constriction in response to repeated pulses of blue that occurred between the different protocol sequences was enhanced in summer compared to winter. This adaptive change in pupil responses to blue stimulation was related to children’s axial lengths, but only in the summer. In the summer months, shorter eyes experienced a greater change in pupillary constriction to blue lights after repeated stimulation compared to longer eyes. A more detailed multivariate examination of pupillary dynamics showed that shorter eyes also showed faster pupillary escape during red light stimulation in the summer months. Also in the summer, shorter eyes showed slower re-dilation following stimulus offset of either color. In the winter months, only re-dilation following red stimulus offset was associated with axial length; shorter eyes re-dilated more slowly than longer eyes with light offset, but only following red stimulation.

Prior study of the relationship between the change in pupillary responses to repeated light stimuli and refractive error in adults using a similar protocol showed some similar results^37^. Non-myopic adults had slower re-dilation following blue stimulus offset, similar to the shorter-eyed participants here. However, in adults, the dilation decay coefficient following red stimulus offset was not significant, and neither red-blue differences nor decay coefficients for constriction were studied. It is worth noting that the sign for the coefficients for ΔRed and blue-offset dilation decay in the previous multivariate analysis in adults are consistent with those for children in the current study. Other studies using different protocols have found no connection between melanopsin-driven pupillary responses and refractive error^44–46^. These studies used shorter protocols with fewer exposures to light stimuli, suggesting that there is no relationship between basal dark-adapted melanopsin-driven pupil responses and refractive error or axial length. Our results instead point to a relationship between axial length and certain adaptive changes in pupil responses to repeated red and blue stimuli. The pupillary responses to the stimuli in the single-color sequences, which occurred later in the protocol, were significantly different than the responses to these same stimuli when applied in the initial alternating sequence. Across the testing sequences (single color compared to initial alternating sequence), pupils constricted more in response to blue than to red and this difference was enhanced in summer (Fig. 3C and D).

Given that light exposure is well established to elevate retinal dopamine levels^20,23^, an intriguing hypothesis is that the changes in the pupil responses to repeated stimuli, seen here and in previous work^37^, relates to effects of dopamine on ipRGCs that contribute to the pupillary light reflex through their input to the olivary pretectal nucleus. In this proposed mechanism, the initial two-minute test in the protocol (consisting of the alternating red and blue light pulses) drives retinal dopamine release through stimulation of both transient and sustained dopaminergic amacrine cells^31^. Dopamine has been shown to enhance melanopsin-driven responses in ipRGCs through its effect on internal cyclic AMP concentrations^47,48^. An enhancement of the melanopsin-driven contribution to the pupillary light reflex in the presence of elevated retinal dopamine is consistent with the finding here of greater pupil constriction to the blue light pulses applied later in the protocol. If this proposed mechanism is correct, our results would suggest that there is more dopamine release (or a greater melanopsin-driven response to similar dopamine levels) in retinas of shorter eyes (correlated with non-myopic refractive error) than in longer eyes (correlated with myopic refractive error). The finding that this relationship is stronger in the summer months also supports this hypothesis. Greater ambient light exposure prior to testing could also contribute to increased retinal dopamine, further potentiating these responses. Light exposure history was not monitored in participants prior to testing in this study. However, it is reasonable to assume that summer provides more opportunity for light exposure and that children will take advantage of time off from school to engage in more outdoor activities^29^. Summer days are up to 6 h longer than winter days in Columbus, Ohio.

The analysis in this study focused on axial length rather than refractive error, differing from previous work^37,44–46^. Axial length was a better variable to consider due to its satisfaction of the normality assumptions for parametric statistics, an assumption refractive error did not meet. Additionally, due to the recruitment procedure for this study, cycloplegic measures were not possible, making axial length a more reliable variable^49^. Finally, it is possible that axial length is a more relevant measure in the pediatric population, as some emmetropic eyes may become myopic in the future.

While this study may add to understanding of the relationship between adaptive changes in pupillary responses with ocular axial length in children, there are some limitations to its results. First, the season in which participation in the study took place may not correlate directly with light exposure before the sessions, limiting the ability to make firm conclusions about the relationship between ambient light exposure and pupillary responses. Direct measure of light exposure has shown that myopic children have lower light exposure than non-myopic children and that greater daily light exposure is related to slower axial growth over a period of 18 months^50,51^. Study of direct measures of light exposure and pupillary responses in children as a function of refractive status did not reveal significant effects, though the previously described shorter testing protocol was used^35^. Future study should consider direct measure of light exposure as a covariate for pupillary responses during the longer pupillary protocol described here in children as a function of axial length and refractive status, as has been done in adults^37^. Testing the same children in the summer and winter months would also allow for more direct analysis of how pupillary responses vary by season. Additionally, the cross-sectional nature of this study establishes only a correlation between axial length and pupillary responses. A longitudinal study of pupillary responses of non-myopic children as they either become myopic or remain non-myopic would aid in the understanding of whether these adaptive response differences exist prior to the development of refractive error, or if they develop along with refractive error.

Finally, the winter cohort studied here was too small to provide sufficient statistical power to find an association between axial length and pupil responses of the same magnitude as in the summer. However, despite this limitation, this study shows an association between shorter axial lengths in children and changes in pupillary responses to repeated red and blue light stimuli in the summer months, potentially driven by light-induced release of retinal dopamine. While we cannot rule out that this association may exist in winter as well, the data do suggest that the relationship is enhanced in the summer with its greater light exposure. This finding may be valuable in understanding the mechanisms underlying myopia development and the environmental risk factors that contribute to it.

## Data Availability

The datasets analyzed for this study are available from the corresponding author on reasonable request.
